# The Frequency, Contributing and Preventive Factors of Harassment towards Health Professionals in Iran

**Published:** 2015-07

**Authors:** Masoud Fallahi Khoshknab, Fatemeh Oskouie, Nahid Ghazanfari, Fereshteh Najafi, Zahra Tamizi, Shahla Afshani, Ghazal Azadi

**Affiliations:** 1 Department of Nursing, University of Social Welfare and Rehabilitation Sciences, Tehran, Iran;; 2 Center for Nursing Care Research, Department of Nursing, School of Nursing and Midwifery, Iran University of Medical Sciences, Tehran, Iran;; 3 Department of Nursing, Razi Psychiatric Hospital, Tehran, Iran;; 4 Department of Nursing, University of Social Welfare and Rehabilitation Sciences, Tehran, Iran;; 5 Deputy of Nursing, Ministry of Health and Medical Education, Tehran, Iran;; 6 Department of Statistics, School of Mathematical Sciences, Shahid Beheshti University, Tehran, Iran

**Keywords:** Health professional, Racial harassment, Sexual harassment, Workplace violence

## Abstract

**Background:**

There are high levels of sexual harassment in health care systems. Also, workplace violence occurs against ethnic and racial minorities. This study aimed to identify the frequency of and the factors contributing to and preventing sexual and racial harassment in the workplace towards health professionals in Iran.

**Methods:**

This cross-sectional study was conducted on 6500 out of 57000 health workers who were selected by multistage random sampling from some teaching hospitals in Iran. Data were collected using the questionnaire of “workplace violence in the health sector” developed by the International Labor Organization, International Council of Nurses, World Health Organization, and Public Services International.

**Results:**

According to the findings, the frequencies of sexual harassment and racial harassment were, respectively, 4.7% and 12% for the 12 months prior to the study (2011). Among healthcare workers, nurses reported the highest rate of violence. The most important contributing factors in sexual and racial harassment were lack of security facilities (45.8%) and people’s ignorance of employees’ tasks (55.7%). The presence of security force, safety measures in the wards, and guards were noted as the most important preventive factor to harassment.

**Conclusion:**

Based on the results, the frequency of sexual and racial harassment is low, which can be attributed to underreporting due to cultural sensitivity or fear. So, identifying the reasons for refusal to report harassment, developing a clear mechanism for reporting and providing the necessary trainings to health workers are essential in order to deal with harassment.

## Introduction

Today, workplace violence is one of the most important issues in healthcare systems and is considered as an important health priority by the World Health Organization (WHO), International Council of Nurses and the Public Services International.^1-4^ According to the definition of WHO, workplace violence is classified into a variety of types, including physical, psychological, sexual and racial.^5^

The possibility of experiencing workplace violence is 16 times greater among healthcare workers and 3 times greater among nurses as compared to other health employees.^6^^,^^7^ Although the frequency of sexual and racial harassment is lower than other types of violence, these figures should not be underestimated, as not only will they affect the physical and mental health of victims, but also they will have adverse effects on the patients, family members of victims and their coworkers.^8^

Sexual harassment (SH) is defined as any type of unwelcome sexual behavior that occurs in verbal, non-verbal, physical, mental or visual forms^9^ and is accompanied by insult, humiliation or threat to the victim’s health.^8^ SH is a major problem at workplace, causing humiliation and embarrassment and will damage the performance of a health team. In addition, SH leads to serious physical, mental and emotional problems and can have negative effects on productivity, turnover, absenteeism, or legal conflicts.^9^

Racial harassment (RH) is defined as any threatening behavior in relation to race, color, language, nationality, religion, or in relation to certain minority issues, birth or other conditions affecting the dignity of women and men in the workplace.^10^

Chen et al. indicated that the prevalence of SH and RH among health workers in a psychiatric hospital were 9.5% and 4.5%, respectively.^11^ In another study,^8^ the prevalence of SH was 51.2% among nurses. The most common forms of SH were verbal (46.6%), visual (24.8%), psychological (20.9%), physical (20.7%), and non-verbal (16.7%).The results of a systematic review showed that the frequencies of SH and RH among health workers in Iran were between 1.07%-9.5% and 12%-20.7%, respectively.^12^ In some studies, more than half of the health workers had experienced SH at least once, as the nature of the nursing profession is working in close contact with patients and medical workers.^13-15^ Studies show that young women and those who have less education and work experience are more likely to be the victims of SH.^14^^,^^16^

The most common form of SH is verbal and is usually committed by men. Also most victims do not report SH.^17^^,^^18^

It is noteworthy that due to cultural and social sensitivity in the country, few studies have investigated the frequency of SH and RH in the workplace compared to other types of harassment. Because few studies have been conducted in this area in Iran and there in no clear image of the frequency and contributing factors of SH and RH, this study was conducted in order to investigate the frequency of and the contributing and preventive factors in SH and RH towards health professionals in some teaching hospitals in Iran. 

## Materials and Methods

This cross-sectional study was conducted in 2011 in some teaching hospitals in Iran. The study population includes physicians, nurses, midwives, nurse aides and paramedical personnel that comes to 57000 according to the latest statistics of the Ministry of Health and Medical Education in 2011. Inclusion criteria were as follows: (i) being currently employed in a teaching hospital (ii) having at least 1 year of experience. Exclusion criterion was a participant’s decision to leave the study. 

The Participants were selected by multistage random sampling in three stages. In the first stage, all of the provinces were included in this study (four provinces excluded due to administrative issues). Sample size of each province was calculated according to the proportion of the health care professionals. In the second stage, hospitals were selected by cluster random sampling based on geographic areas. Therefore, 135 teaching hospitals were selected. In the last stage, samples were selected by random sampling based on sample size in each hospital. Sample size was estimated by the formula: (Z=1.96; α=0.05)

$$m={\left(\frac{1.96}{0.01}\right)}^{2}\left(0.75\right)\left(0.25\right)=7203$$

$$n=\frac{\left(7203)(65000\right)}{7203+65000}=6485$$

m=1-α2Zd2(p)(1-p)

$$n=\frac{\left(M)(total\right)}{M+total}$$

The Study instrument was the international questionnaire of “workplace violence in the health sector” developed by the International Labor Organization, International Council of Nurses, World Health Organization, and Public Services International in 2003. The questionnaire consists of five parts: the first part includes 21 questions about personal and workplace issues, the second part includes 17 questions related to physical violence, the third part includes 37 questions about psychological violence in the workplace, including verbal abuse, bullying/mobbing, SH and RH, the fourth part includes 8 questions about health section, and the fifth part includes 3 open-ended questions about the views of participants about workplace violence.^10^ The questionnaire was translated from English to Persian by a person who was proficient in both languages and then back-translated from Persian to English by another person who was also proficient. The content validity of the questionnaire items was assessed by a committee including eleven experts. The items were modified according to the experts’ recommendations. In order to evaluate the reliability, the questionnaire was completed by 180 health professionals twice with a 15 day interval, and its reliability was confirmed, r=0.71. In this paper, due to the large amount of the data and number of the tables, only the results of the third part are reported.

This study was approved by the Ethics Committee of Tehran University of Social Welfare and Rehabilitation Sciences (Ethics Committee Approval Number: 14/86). Also, before data collection, entrance permission to hospital units was obtained. Then, in addition to explaining the objectives of the study to the participants, the researchers asked for their written informed consent. The anonymity of the participants and voluntary participation in the study were ensured.

Data were analyzed based on descriptive statistics (means, standard deviations, and frequencies) using SPSS software version 13. 

## Results

The response rate was 90.36% (5874 people). The average age of the participants was 34±8.5 years. Most of the participants were female (n=4790; 82.71%) and married (n=4212; 72.7%).The majority of the participants were registered nurses (n=4505; 78.5%). The average work experience was 10.35±7.4 years. The majority of the participants reported that they worked in different units of the hospitals overtime (n=3937; 75.05%). More than half of the participants reported that they worked between 7 p.m. and 7 a.m. (n=3269; 58.5%). Almost all of the participants reported that they had direct contact with patients during their work (n=5471; 97.6%). More than half of the participants (n=2539; 60.6%) pointed out that there was no protocol for reporting workplace violence. The majority of the participants (n=3767; 85.1%) had not been in training a program for management and prevention of violence in the workplace (Table1).

**Table 1:** Demographic and occupational Characteristics of participants (N=5874)

**Variable**	Number *	**Percentage**
Gender		
Male	1001	17.3
Female	4790	82.7
Age(years)		
<30	1544	28
30-40	2414	44
41-50	1337	24.3
51-60	199	3.5
>60	9	0.2
Marital Status		
Single	1574	27.3
Married	4212	72.7
Profession		
Nurse	4505	78.5
Physician	43	0.75
Midwife	239	4. 15
Nurse aide	619	10.8
Paramedic	337	5.8
Work between 7p.m. and 7 a.m.		
Yes	3269	58.5
No	2322	41.5
Work areas		
One area	1310	25
Multiple areas (overtime)	3937	75
Professional experience in health care services(years)		
0-10	3215	58.8
11-20	1673	30.6
21-30	576	10.6
Employment status		
Full time	2344	42.9
Part time	680	12.5
Overtime	2433	44.6
Direct patient/client contact		
Yes	5471	97.6
No	132	2.4
Sex of the patients that health workers most frequently work with		
Male	599	10.8
Female	844	15.1
Male and Female	4120	74.1
Presence of security guards in the wards		
Yes	2036	37
No	3462	63
Workplace has been highly dangerous		
Always	1855	32.7
Sometimes	2746	48.5
Never	1069	18.8
Presence of Protocols for reporting workplace violence		
Yes	1653	39.4
No	2539	60.6
Presence of training programs related to incident management		
Yes	660	14.9
No	3767	85.1

*The sum may be less than the total number of participants because of missing data.

According to the findings, the frequencies of SH and RH during the year prior to the study were 4.7% and 12%, respectively. Also, 2.1% of the participants had experienced both types of harassment. Due to cultural sensitivity, the participants were not asked about the details of their SH; but regarding RH, the findings showed that 24.3% of harassment was due to ethnicity issues, 18.6% was due to racial issues, 18.8% was due to language differences, 8.3% was due to religious issues, 14.6% was due to birth place, and 15.4% of the participants reported a combination of factors involved in their harassment. 40.7% of SH and 46.3% of RH occurred in the morning shifts. Nurses were the main victims of such violence. Furthermore, Patients’ families and relatives were the main perpetrators of the violence. More than half of SH(n=167;76.9%) and RH(n=353;66.1%) offenders were men. More than half of the participants had not reported the harassment. The most important reason for not reporting was the fact that it was considered useless (Table 2).

**Table 2:** Frequency of sexual and racial harassment and reactions of health care workers (N=5874)

**Variable**	**SH ** N*(%)	**RH** N*(%)
Exposure to violent incidents in the past 12 months		
Yes	242 (4.7)	603 (12)
No	4934 (95.3)	4437 (88)
Time of violent incident occurrence		
07.00a.m.-3.00 p.m.	88 (40.7)	231 (46.3)
3.00 p.m.-11.00 p.m.	48 (22.2)	136 (27.2)
11.00 p.m.-07.00 a.m.	80 (37.1)	132 (26.5)
Gender of perpetrators		
Male	167 (76.9)	353 (66.1)
Female	50 (23.1)	181 (33.9)
Responsible persons for violent incidents		
Patient	63 (33)	78 (18.4)
Relatives of patients/clients	110 (57.6)	204 (48.1)
Staff members	12 (6.3)	118 (27.8)
Managers/supervisor	6 (3.1)	24 (5.7)
Exposure to violence based on the profession		
Nurse	173 (72.7)	448 (75.2)
Physician	3 (1.26)	9 (1.5)
Midwife	3 (1.26)	18 (3)
Nurse aide	52 (21.84)	87 (14.6)
Paramedic	7 (2.94)	34 (5.7)
Reactions of participants toward violence		
Took no action	163 (67.3)	407 (67.4)
Tried to pretend it never happened	50 (20.66)	113 (18.73)
Told the person to stop	107 (44.2)	252 (41.79)
Tried to defend themselves	43 (17.76)	116 (19.23)
Told friends/family	13 (5.37)	46 (7.62)
Told a colleague	64 (26.44)	144 (23.88)
Sought counseling	17 (7.02)	30 (4.97)
Sought help from union	34 (14.04)	77 (12.76)
Completed incident/accident form	0 (0)	10 (1.65)
Completed a compensation claim	3 (1.23)	8 (1.32)
Reporting the incident		
Yes	101 (47.2)	236 (43.4)
No	113 (52.8)	308 (56.6)
Reasons for not reporting the incident		
It was no important	24 (9.9)	80 (17.9)
Felt ashamed	25 (10.3)	39 (8.76)
Felt guilty	2 (0.8)	7 (1.57)
Afraid of negative consequences	17 (7.02)	46 (10.3)
Useless	80 (33.05)	240 (53.9)
Did not know who to report to	10 (4.13)	33 (7.4)
Action taken with regard to the incident occurred		
Yes	60 (28.2)	123 (23.2)
No	153 (71.8)	408 (76.8)
Authorities who took action		
Head nurse	26 (33)	56 (33.5)
Management	43 (54.4)	78 (46.8)
Police	10 (12.6)	33 (19.7)
Satisfaction with the action taken		
Very dissatisfied	128 (67)	319 (65.1)
Dissatisfied	19 (10)	66 (13.5)
Moderately satisfied	31 (16.2)	77 (15.7)
Satisfied	13 (6.8)	28 (5.7)
Very satisfied	0 (0)	0 (0)

*The sum may be less than the total number of participants because of missing data.

According to the results, the most important contributing factors in SH and RH were lack of security facilities (n=111; 45.8%) and people’s ignorance of employees’ tasks (n=336; 55.7%). Moreover, the presence of security forces and safety measures in the wards were noted as the most important deterrents to harassment. Participants could choose more than one option (Table 3).

**Table 3:** Contributing and preventive factors in sexual and racial harassment

**Contributing factors**	**SH (n=242)** **Number (%)**	**RH (n=603)** **Number (%)**
Drug or alcohol use by patients	64 (26.4)	134 (22.2)
Staff shortage in the ward	83 (34.2)	193 (32)
Patient’s judicial and legal Issues	16 (6.6)	56 (9.2)
Lack of security facilities	111 (45.8)	260 (43.1)
Patient’s death	32 (13.2)	94 (15.5)
Patients’ ignorance of employees’ tasks	110 (45.4)	336 (55.7)
Delays in aid	21 (8.6)	76 (12.6)
Lack of training program for preventing violence	48 (19.8)	126 (20.9)
Prolonged stay of the patients in the ward after discharge	38 (15.7)	80 (13.2)
Interval between hospital admission and diagnosis of the patient’s disease	24 (9.9)	42 (7)
Gathering of high-risk patients in one room	35 (14.4)	81 (13.4)
**Preventive factors** **in workplace violence**		
Safety measures in wards	115 (47.5)	319 (53)
Presence of police force	124 (51.2)	315 (52.2)
Presence of guards	112 (46.2)	286 (47.4)
Reporting harassment	68 (28)	173 (28.7)
Guidelines to deal with harassment	71 (29.3)	205 (34)
Employees’ training	78 (32.2)	230 (38.1)
Separating addicted patients from other patients	62 (25.6)	159 (26.4)

## Discussion

The results of the study showed that the frequencies of sexual and racial harassment during the year prior to the study were 4.7% and 12%, respectively. Hesketh et al. pointed out that sexual harassment against nurses was between 8.6% and 19.5% in different hospital units in Canada.^19^ Also, in another study in England, more than 52% of the surveyed women had experienced some form of sexual harassment,^20^ which indicates a higher frequency of sexual harassment in international studies compared to the current study. This finding can be possibly due to higher rate of reporting, cultural differences as well as differences in workplaces in these countries. It seems that in some countries, these types of harassment are reported less often due to cultural sensitivity.

The frequency of racial harassment was reported to be 26% in America.^21^ The frequencies of sexual and racial harassment among psychiatric nurses working in one of the psychiatric hospitals in Tehran were 5.5% and 19.1%, respectively.^22^ These findings are consistent with those of the current study. According to the findings of Talas et al., unwanted sexual jokes, stories, questions or words were the most common forms of sexual harassment.^23^ In another Korean study, verbal abuse was the most common form of sexual harassment.^9^ In the current study, questions about the details and the type of sexual harassment committed in the workplace were removed due to cultural sensitivity.

The results showed that the main factor in racial harassment was ethnic issues. In America, the rate of racial harassment was not higher among black employees than white ones.^21^ None of the types of racial harassment were noticed in any of the studies in Iran.

According to the results, the highest rate of harassment had occurred during the morning shift. Suhaila et al. reported that 57.1% of sexual harassment incidents occurred during the night shift,^8^ and Lee et al. reported the highest rate of sexual harassment in the afternoon shift,^9^ which disagrees with the results of the current study. The discrepancy may be attributed to underreporting in these shifts.

In the current study, the highest rate of harassment was committed by patient’s family members, which corresponds with other studies.^8^^,^^9^^,^^12^^,^^23^

In the present study, more than half of the participants had not reported harassment because they mostly thought reporting was useless. According to the results of a study in Malaysia, the main reason for failure to report was the lack of a mechanism for reporting, lack of appropriate methods of reporting, and not knowing to whom one should report,^8^ which findings disagree with the findings of the current study. This discrepancy may be attributed to cultural differences affecting the report of harassment, especially sexual harassment.

Most of the participants had not taken training courses for controlling harassment, which is consistent with the results of other studies.^22^^,^^23^

According to the findings, the most important contributing factors in workplace violence were lack of security facilities and people’s ignorance about tasks of employees. Also, the presence of security forces and safety measures in the wards were noted as the most important preventive factors. The results of other studies show that lack of timely security facilities and the nurse to patient ratio were the most important factors contributing to harassment,^24^ while the presence of guards, safety measures in wards and employee training programs were the most important preventive factors,^22^ which results are consistent with the findings of the current study.

Some of the limitations of this study included the self-reporting method and the physical and psychological conditions of the respondents that could have affected the way they answered. Also, the nature of this type of harassment left some questions unanswered which can lead to underreporting. Finally, data were collected retrospectively, which might lead to recall bias. 

## Conclusion

This study is the first extensive research in this field in Iran. The findings showed that the frequency of sexual and racial harassment in Iran is lower than in some other countries, which can be attributed to underreporting because of cultural sensitivity or fear of reporting. However, due to the negative consequences resulting from this type of harassment, proper practical measures should be taken, including the development of a clear mechanism for reporting, managers’ dealing with cases of harassment, and providing the necessary training programs for health professionals on how to deal with harassment. 
